# Contribution of snowfall from diverse synoptic conditions in the Catskill/Delaware Watershed of New York State

**DOI:** 10.1002/joc.6043

**Published:** 2019-03-11

**Authors:** Zachary J. Suriano, Daniel J. Leathers, Dorothy K. Hall, Allan Frei

**Affiliations:** ^1^ Department of Geography & Geology University of Nebraska Omaha Omaha Nebraska; ^2^ Department of Geography University of Delaware Newark Delaware; ^3^ Earth System Science Interdisciplinary Center University of Maryland College Park Maryland; ^4^ Cryospheric Sciences Laboratory NASA/GSFC Greenbelt Maryland; ^5^ Department of Geography, Hunter College City University of New York New York City New York

**Keywords:** lake‐effect, New York City, Nor'easter, snow, synoptic classification, water resources

## Abstract

Snowfall in the six basins of the Catskill/Delaware Watershed in south‐central New York State historically contributes roughly 20–30% of the water resources derived from the watershed for use in the New York City water supply. The watershed regularly experiences snowfall from three distinctive weather patterns: coastal mid‐latitude cyclones, overrunning systems, and lake‐effect or Great Lakes enhanced storms. Using synoptic weather classification techniques, these distinct regional atmospheric patterns impacting the watershed are isolated and analysed in conjunction with daily snowfall observations from 1960 to 2009 to allow the influence of each synoptic weather pattern on snowfall to be evaluated independently.

Results indicate that snowfall‐producing events occur on average approximately 63 days/year, or once every 4 days during the October–May season, leading to an average of 213 cm/year of snowfall within the watershed. Snowfall from Great Lakes enhanced storms and overrunning systems contribute nearly equally to seasonal totals, representing 38 and 39%, respectively. Coastal mid‐latitude cyclones, while producing the highest amount of snowfall per event on average, contribute only 16% to the watershed average total snowfall. Predicted climate change is expected to impact snowfall differently depending on the specific atmospheric pattern producing the snow. As such, quantifying the contribution of snowfall to the watershed by synoptic pattern can inform future water management and reservoir operation practices for the New York City Water Supply Management System.

## Graphical abstract

The New York City water supply derives nearly 90% of its water from the Catskill/Delaware Watershed in south/central New York State. Snowfall accumulations in this region are common and occur under a variety of synoptic‐scale conditions, including those shown in the figure. By analysing their relative contributions to the regional snowfall total, further strides are made in quantifying snow and water resource variability for the present, and towards the future.

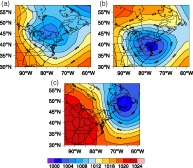

## 1 INTRODUCTION

Snowfall is an important component of the New York City Water Supply System (NYCWSS) that supplies water for the consumption and sanitation needs of roughly 9 million people in New York City (NYC; Matonse *et al.*, 2011). The NYCWSS derives approximately 90% of its water from the six basins of the Catskill/Delaware Watershed (CDW), located in the Catskill Mountains of south‐central New York State (Matonse *et al.*, 2013). The Cannonsville subbasin, situated along the western boundary of the CDW, has historically contributed 50% of the total water supply and is the second largest reservoir used for NYC water needs. Within the CDW, 20–30% of the total annual precipitation is snowfall (Frei *et al.*, 2002; Pradhanang *et al.*, 2011), which may be produced under a variety of atmospheric conditions.

Due to the location of the CDW in south‐central New York State and in the northeast United States in general, the watershed is subject to three general categories of atmospheric conditions leading to snowfall: coastal mid‐latitude cyclones, overrunning systems, and lake‐effect or Great Lakes enhanced patterns (e.g., Karmosky, 2007). The physical processes that induce snowfall during each synoptic situation are different and in‐turn impact the magnitude and spatial distribution of snowfall across the region. As such, it is important to understand the relative contributions to total CDW snowfall from each category and investigate if changes are occurring. Under warmer climates, such as those already observed globally (IPCC, 2013), conditions are less likely to support frozen hydrometeors (see Krasting *et al.*, 2013). While ultimately both liquid and solid precipitation in the CDW will eventually recharge its reservoirs, there is often a delay in the runoff associated with snowfall until the spring months when meteorological conditions are more suitable for ablation. Identifying how much snow, when that snow accumulates, and if those features are changing under various conditions is valuable in informing water management practices, particularly in the cold season. Thus, an investigation into snowfall under various conditions, independent of total precipitation, is warranted.

To identify the atmospheric conditions leading to snowfall across a region, synoptic classification techniques have been used in prior studies (Ellis and Leathers, 1996; Leathers and Ellis, 1996; Karmosky, 2007; Suriano and Leathers, 2017a; 2017b). These techniques are used to classify daily meteorological events into distinct synoptic types to enable evaluation of the atmosphere's influence on the underlying surface, and allow for the frequency and influence of the synoptic‐scale patterns to be evaluated (Yarnal, 1993; Sheridan and Lee, 2014). Each synoptic weather type produces a different spatial pattern and magnitude of snowfall in the CDW, where long‐term changes in the intensity and frequency of these synoptic weather types can alter the snow climatology and influence water resource management practices.

In this study, an eigenvector‐based synoptic weather classification is conducted to generate a daily synoptic calendar of the atmospheric conditions influencing the CDW from 1960 to 2009. The synoptic weather types created by a temporal synoptic index (TSI; Kalkstein and Corrigan, 1986) are then qualitatively grouped into one of three general categories of coastal mid‐latitude cyclones, overrunning systems, and Great Lakes enhanced pattern; the resulting snowfall associated with each is examined. This approach allows for the influence of each synoptic category on snowfall to be determined independently, and thus to quantify their respective relative contribution of total snowfall.

## 2 DATA AND METHODS

### 2.1 Snowfall data

Daily snowfall data are obtained at observation stations within the CDW from the Global Historical Climatology Network (GHCN), available at the National Centers for Environmental Information (NCEI; http://www.ncdc.noaa.gov). Within the CDW, daily surface observations are relatively sparse both spatially and temporally, with many observation stations reporting intermittently over the last 70 years and with station availability dropping in recent years. To be included in this analysis, stations must be located within the geographic bounds of the watershed and have at least 90% non‐missing data over the period 1960–2009. This period was chosen to maximize the number of observation stations while maintaining a climatological perspective. These criteria result in only two stations being available for analysis: Slide Mountain, NY (GHCN: USC00307799) and Arkville 2W, NY (GHCN: USC00300254).

To increase the number of stations available for analysis, observation stations in the same geographic area (<10 km) were evaluated for the potential to merge into a single station, similar to Frei and Kelly‐Voicu (2017). For a merger to be considered, the stations in question needed to be less than 10 km from each other, exhibit less than a 20% difference in elevation, and contain a period of overlapping records. Furthermore, during the periods of overlap, data from the stations in question needed to be significantly correlated, as determined by ordinary least‐squares regression (*p* < .01), while also not exhibiting a systematic jump in snowfall values. Systematic jumps in snowfall were evaluated using a Student's *t* test, where a merger was considered if the *t* test showed the sites’ snowfall data were not significantly different. If two (or more) stations passed all of these criteria, snowfall data from one station was used to populate the missing snowfall data in the other. In the event both stations reported snowfall on the same day, the average of the stations was used. Merged stations must also pass the 90% non‐missing data threshold for consideration. Two further locations were added to the analysis: Delhi, NY and Walton, NY. Delhi was merged from three stations: Delhi 2 SE, NY (GHCN: USC00302036), Delhi 1.5 NNE, NY (GHCN: US1NYDL0005), Lake Delaware, NY (GHCN: USC00304525), while Walton, NY was merged from two stations: Walton, NY (GHCN: USC00308936) and Walton 2, NY (GHCN: USC00308932)). The location and elevation of the observation stations are shown in Table 1.

**Table 1:** Elevation and location information for each of the observation stations used in the study. Delhi 2 SE, Delhi 1.5 NNE, and Lake Delaware stations are merged into a single Delhi station, while Walton and Walton 2 are merged into a single Walton station as described in section 2.1

	Delhi 2 SE	Delhi 1.5 NNE	Lake Delaware	Walton	Walton 2	Arkville 2 W	Slide Mountain
Elevation (m)	445.0	462.1	448.1	378.0	451.1	399.3	807.7
Lat/Lon (°)	42.3, −74.9	42.3, −74.9	42.3, −74.9	42.2, −75.1	42.2, −75.1	42.1, −74.7	42.0, −74.4

**Figure 1:**
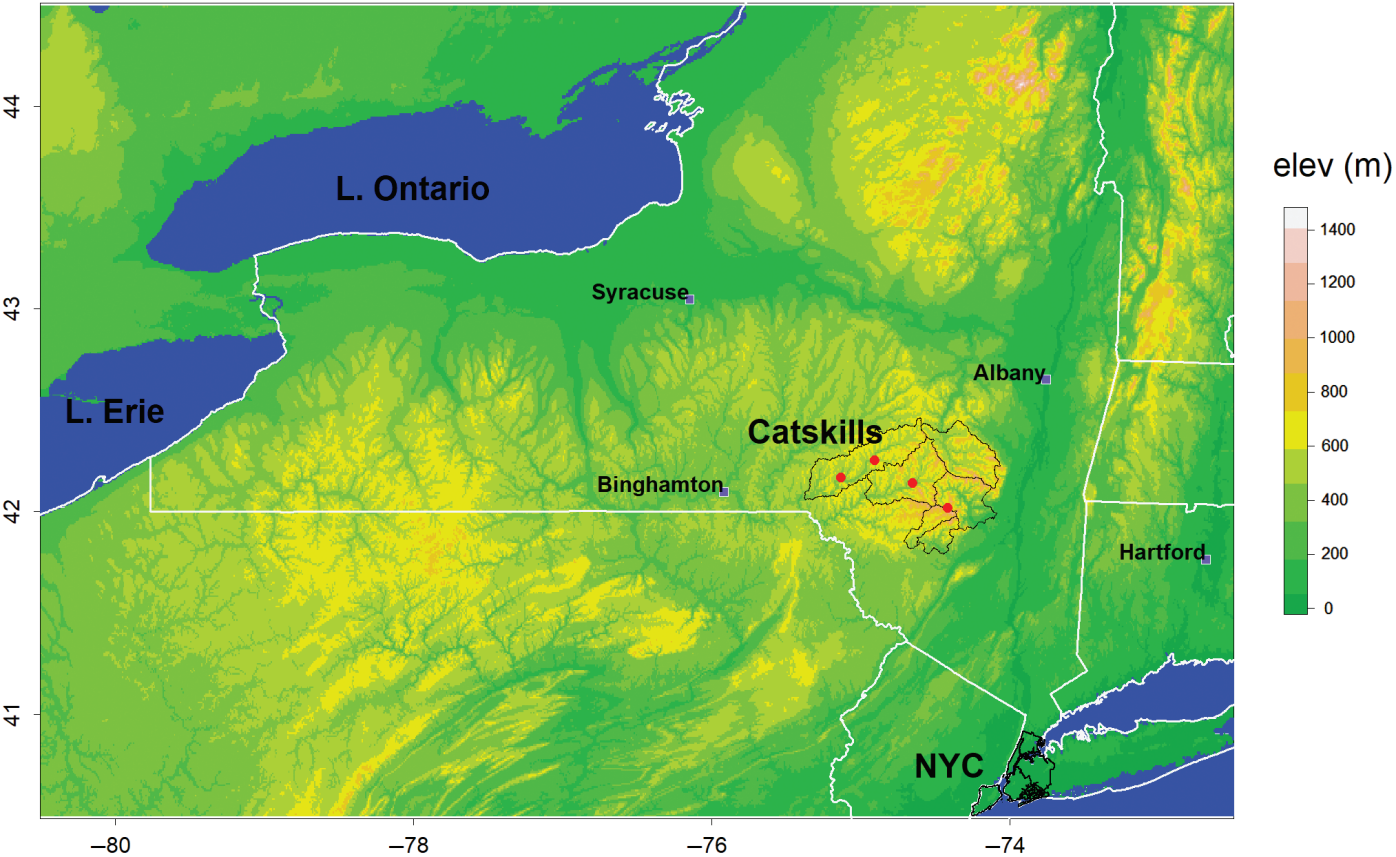
Topographic map (m) depicting the CDW (black lines) in south‐central New York State. The four observation stations used in the analysis represented as red dots within the watershed (from left to right: Walton, Delhi, Arkville, Slide Mountain)

In total, four locations within the CDW are available for analysis: Delhi, Slide Mountain, Arkville, and Walton, with three of the six sub‐basins within the CDW represented (Figure 1). The Cannonsville sub‐basin contains two stations (Walton and Delhi) and has been identified as an important contributor to the watershed's hydrology by intercepting a majority of snow travelling from the west (Hall *et al.*, 2017; Hall *et al.*, 2018). The Pepacton and Ashokan sub‐basins each contain one observation station, while the three smallest sub‐basins (Neversink, Rondout, and Schoharie) are not represented.

### 2.2 Temporal synoptic index

To determine the synoptic‐scale atmospheric patterns that lead to snowfall in the CDW, a TSI, described in Kalkstein and Corrigan (1986), is utilized. The TSI is a multivariate, eigenvector‐based, synoptic weather classification procedure where a principal component analysis (PCA) is conducted, followed by a cluster analysis. The PCA is used for data decomposition, resulting in a new set of orthogonal variables for clustering. The objective is to create relatively homogenous clusters of weather patterns in which within‐cluster variance is minimized while between‐cluster variance is maximized, such that the influence each cluster (i.e., synoptic type) has on snowfall can be isolated individually.

The TSI is advantageous in that sub‐daily meteorological observations taken at a single observation station can provide a robust indication of the synoptic‐scale atmospheric environment over a sub‐continental region, without the need for upper air observations or multiple surface stations (Kalkstein and Corrigan, 1986). The procedure has been successfully used in a variety of climatological and meteorological applications including long‐term climatic change (Kalkstein *et al.*, 1990), aerosol variability and ozone pollution (Kalkstein and Corrigan, 1986; Davis, 1991; Brodie *et al.*, 2017), and snowfall and snow ablation variability (Ellis and Leathers, 1996; Leathers and Ellis, 1996; Karmosky, 2007; Suriano, 2018, among others). While similar procedures, such as self‐organizing maps, may have produced viable results, the TSI is a time‐tested procedure for classifying synoptic‐scale environments.

Meteorological observations of temperature, dew point temperature, sea level pressure, *u* and *v* wind vectors, and cloud cover reported four times daily (0900, 1500, 2100, 0300 UTC) from Hartford, CT (WBAN# 14740, 41.94°N, 72.68°W) are obtained. Hartford is located within 100 km of the CDW and its location is suitable for analysis of the entire northeast region's synoptic‐scale environment, including the CDW. While stations such as Albany, Syracuse, or Binghamton, NY may have also served as reasonable locations for developing a synoptic weather‐type classification, the differences between the TSI at the three locations can be assumed to be minimal for the synoptic‐scale environment. Hartford was ultimately chosen due to its location and low percentage of missing data during the study period (<0.3%).

An unrotated R‐mode PCA is conducted on the meteorological observations (SPSS Inc. 2017) to determine the main modes of variability for each climatological season: autumn (SON), winter (DJF), and spring (MAM). Summer (JJA) is not included due to the lack of snowfall in the watershed during those months. The PCA is conducted seasonally as opposed to over the entire October–April snow season as analysis of a 7‐month period results in much of the explained variance of the PCA being attributed to the annual cycle, limiting the effectiveness of the procedure (Siegert *et al.*, 2017; Suriano and Leathers, 2017a; 2017b).

The PCA reduces the original 24 daily variables (four reporting periods of six variables) into a set of linearly independent components. Only seasonal PCA components with an eigenvalue greater than 1.0 are retained, indicating those components account for more variability than any single original variable. The 1.0 threshold is common practice in synoptic climatology and is confirmed by examining the scree plot. Daily component scores are then calculated for each principal component by multiplying the eigenvector of each component by the original data. Principal component scores represent the importance of a particular component to that day's weather (Kalkstein *et al.*, 1990). Days with similar component scores have similar meteorological conditions, thus can be clustered to categorize days into distinct groups (Leathers and Ellis, 1996). Within‐group average linkage clustering analysis is identified by Kalkstein *et al.* (1987) to be the most appropriate method for synoptic weather type classification due to its differentiation of extreme and more normal days into appropriate clusters. The algorithm for average linkage allows a case to be included within a cluster based on the squared Euclidean distance between the case at hand, and all of the other cases already belonging to the cluster. This cluster analysis is conducted on the unrotated principal component scores with an initial 20‐cluster solution per season. Twenty clusters represent a typical maximum number of solutions and aids in preventing synoptic weather types from being coerced due to the constraints on initial solution numbers. Due to the use of the more appropriate within‐groups average linkage clustering, the TSI conducted in this study is slightly different from that of Kalkstein and Corrigan (1986) which used a Ward's clustering.

The result of the TSI procedure is a daily synoptic calendar where each day from 1960 to 2009 is classified as a specific synoptic weather type (i.e., cluster solution) and can be examined in conjunction with the daily snowfall in the CDW. To aid in visualizing the atmospheric conditions associated with each synoptic weather type spatially across the northeast United States, composite maps are derived depicting the average conditions across all occurrences of each individual weather type. The composites are created for sea level pressure, surface air temperature, and 500‐hPa geopotential heights using the National Center for Environmental Prediction (NCEP)/National Center for Atmospheric Research (NCAR) Reanalysis (Kalnay *et al.*, 1996). Additional atmospheric variables may be composited to further differentiate the atmospheric patterns. It should be noted that precipitation, including snowfall, is not used to define the synoptic weather types.

### 2.3 Climatological analysis methodology

A brief snowfall climatology is initially presented for the watershed where snowfall from each of the four stations is averaged into a single daily watershed‐wide snowfall time series using linear interpolation. To understand the impact synoptic weather types have on snowfall within the CDW, daily snowfall values and the daily synoptic calendar are analysed together. The percentage of the total snowfall attributed to each individual synoptic type is calculated at the four stations and averaged into a watershed‐wide value. Only synoptic weather types that result in at least 1.0% of the watershed‐wide total snowfall are retained for analysis. Twenty‐one (21) synoptic weather types meet this criterion, accounting for 93.3% of the total snowfall across the three non‐summer seasons.

**Figure 2:**
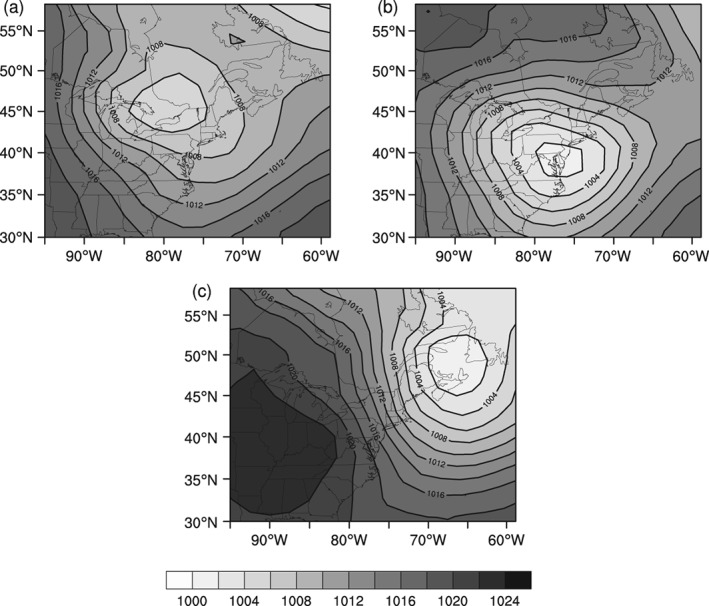
Composite sea level pressure fields (hPa) for representative examples of the three snowfall‐inducing synoptic categories (a) type OR‐1 (overrunning), (b) type CO‐3 (coastal), and (c) type LE‐4 (Great Lakes enhanced). Lighter shades represent lower SLP while darker shades represent higher SLP

Rather than examining the snowfall associated with each of the 21 synoptic weather types individually, the types are qualitatively categorized into three primary patterns that result in snowfall within the CDW: coastal mid‐latitude cyclones, overrunning systems, and Great Lakes enhanced patterns (Figure 2). Coastal mid‐latitude cyclones are commonly, but not exclusively, represented by Nor'easters of either a Miller Type “A” or “B” categories in which a mid‐latitude cyclone tracks northward along the Atlantic coast (Miller, 1946; Kocin and Uccellini, 2004). Overrunning systems are classified as both inland tracking mid‐latitude cyclones and flow regimes other than coastal and Great Lakes enhanced patterns that lead to precipitation in the region (Karmosky, 2007).

Great Lakes enhanced patterns are associated with a Low in maritime Canada and/or a High over the midwest United States that leads to west/northwest flow over the Great Lakes and atmospheric conditions that are generally suitable for the development of lake‐effect snow, as documented in Suriano and Leathers (2017a; 2017b). It is important to note that a Great Lakes enhanced pattern distinction in this study does not necessarily mean the snowfall from these patterns is exclusively lake‐effect snow in the CDW. Lake‐effect snow is primarily a mesoscale process, but the synoptic‐scale atmospheric conditions suitable for lake‐effect development can be detected using the above methodology. Care is taken to differentiate synoptic types in this category that appear to have a higher probability of snowfall considered to be “wrap‐around” snow from a mid‐latitude cyclone, from those suitable for lake‐effect snow (Suriano and Leathers, 2017a) based on the configuration of surface pressure. One synoptic type (OR‐6) is discussed in detail in section 4 as an example of this process. As such, the majority of snowfall from Great Lakes enhanced patterns is considered to be lake‐effect or lake‐enhanced snow, with potentially only small amounts of wrap‐around snow from a mid‐latitude cyclone.

The 21 synoptic weather types are qualitatively placed into the three general categories based on the atmospheric configuration across the northeast United States, depicted in the composited variables, with particular emphasis placed on sea level pressure and wind flow configurations. Additionally, daily composites are examined on days prior to, and after the synoptic pattern influences the CDW to assist in accurate categorization. Snowfall, percent of total snowfall, frequency of snowfall events, and the average magnitude of snowfall per event are examined for each of the three synoptic categories across the watershed to understand the relative contribution to snowfall from each category. These variables are investigated as a 1960–2009 average, inter‐annually, and monthly. To determine if changes to the snowfall associated with these synoptic categories occurred, long‐term trends are examined using ordinary least‐squares linear regression.

## 3 RESULTS

### 3.1 Snowfall climatology

**Figure 3:**
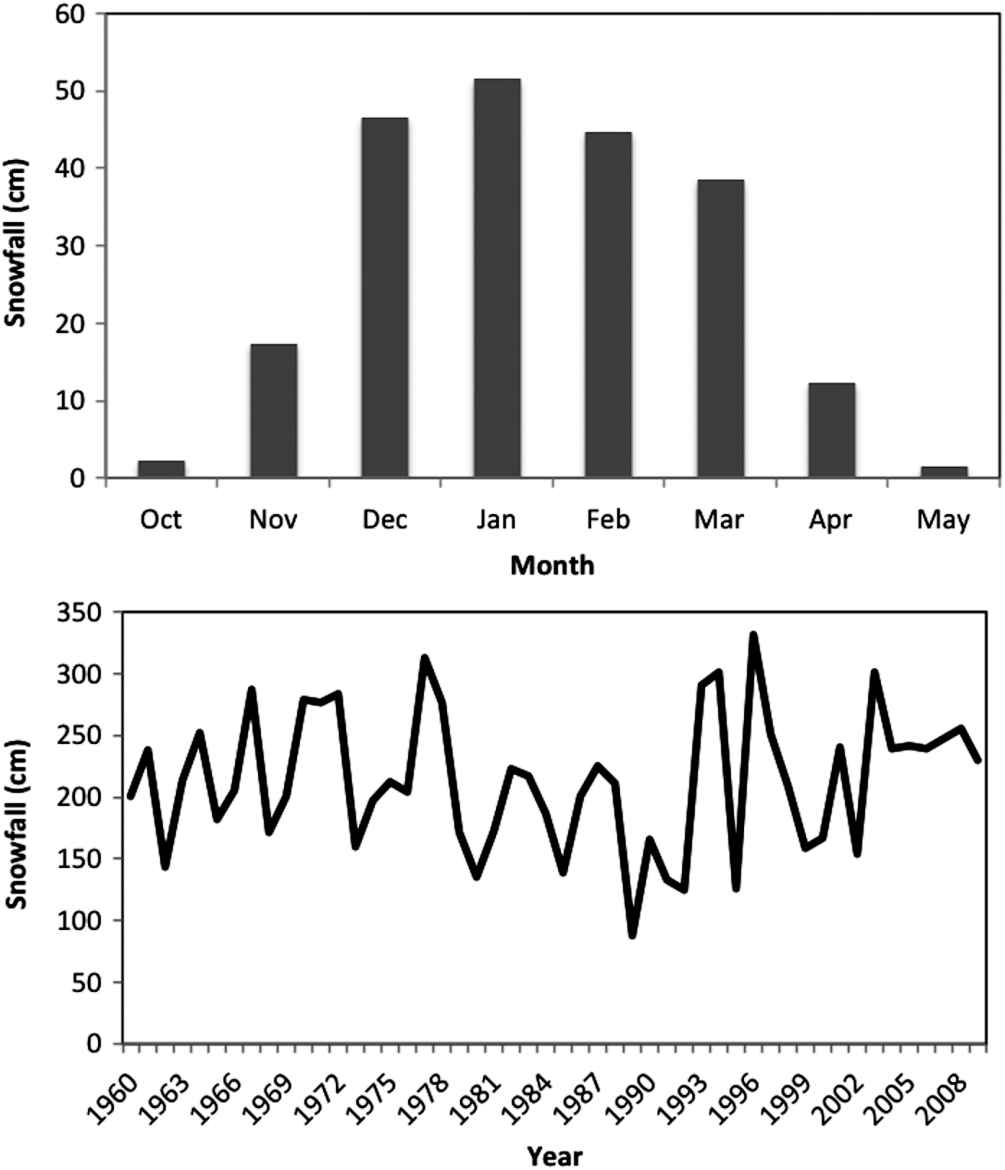
The seasonal cycle of total snowfall (cm) averaged over 1960–2009 for the CDW and the inter‐annual frequency of October–May snowfall

Within the CDW, snow may accumulate from October through May; however, it is most prominent during the months of December, January, February, and March in which each month, respectively, contributes approximately 21.8, 24.1, 20.9, and 18.0% of the seasonal total (Figure 3a). On average, approximately 213.3 cm (±56.1 cm) of snowfall is recorded each season across the watershed, where snowfall totals can vary from less than 88 cm/year to over 330 cm/year (Figure 3b). Snowfall events (days where snowfall is recorded in the watershed) occur roughly 70 days/year (±11.6) and result in an average of 3.0 cm (±0.6 cm) in accumulation per event. Seasonal snowfall does not exhibit a significant long‐term trend from 1960 to 2009.

### 3.2 Synoptic weather typing

**Figure 4:**
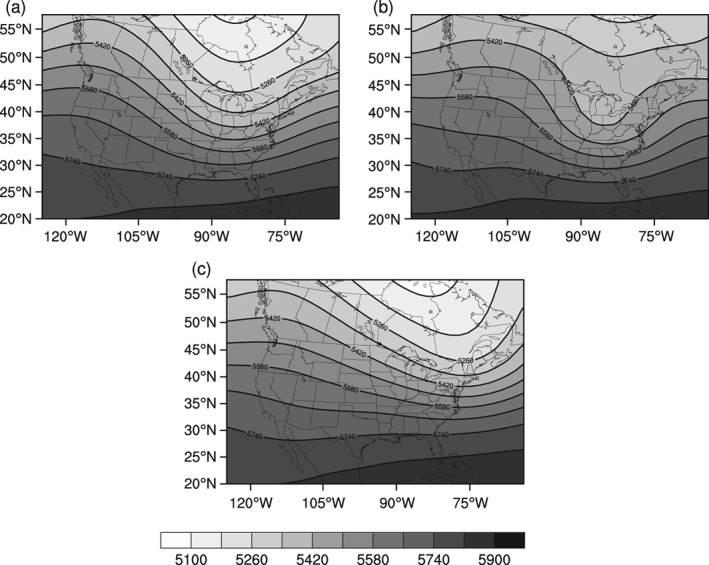
Composite 500‐hPa geopotential height fields (gpm) for representative examples of the three snowfall‐inducing synoptic categories (a) type OR‐1 (inland), (b) type CO‐3 (coastal), and (c) type LE‐4 (Great Lakes enhanced). Lighter shades represent lower 500 hPa heights while darker represents higher heights

The PCA procedure of the TSI results in six principal components during the winter season and five during the autumn and spring seasons with eigenvalues greater than 1.0. Collectively, the principal components respectively explain 78.1, 81.2, and 77.7% of the seasonal autumn (SON), winter (DJF), and spring (MAM) variance. After the cluster analysis, as described in the methodology, only the 21 synoptic weather types that contributed at least 1% of the seasonal total snowfall are analysed further. Average daily meteorological conditions in Hartford, CT for each of the 21 analysed synoptic types are presented in Table 2 along with the assigned classification of synoptic category: overrunning systems (OR), coastal mid‐latitude cyclone (CO), or Great Lakes enhanced pattern (LE). Composite sea level pressure fields for each of the 21 synoptic types are available in the supplementary information. For reference, a representative sea level pressure field for each synoptic category is depicted in Figure 2, where types OR‐1 (Figure 2a), CO‐3 (Figure 2b), and LE‐4 (Figure 2c) respectively represent the overrunning systems, coastal mid‐latitude cyclones, and the Great Lakes enhanced categories. Similarly, Figure 4a–c depicts composite 500‐hPa geopotential height for each of the previously mentioned types (OR‐1, CO‐3, LE‐4).

**Table 2:** Average daily meteorological conditions in Hartford, CT for each of the 21 synoptic weather types that lead to at least 1% of the total snowfall in the CDW

Synoptic type	Temperature (°C)	Dewpoint temperature (°C)	Sea level pressure (hPa)	Wind speed (m/s)	Wind direction (°)	Cloud cover (1/10)	Snowfall per event (cm)	Percent of total snowfall	Number of days with snowfall	Percent snow occurrences
OR‐1	−0.4	−4.8	1003	2.0	297	7.4	3.6	3.5	101	62
OR‐2	−3.9	−11.4	1022	2.7	330	2.5	2.1	4.3	224	45
OR‐3	−8.6	−12.9	1010	3.3	0	9.1	5.8	3.1	56	64
OR‐4	−3.4	−8.2	1023	2.1	352	8.5	2.7	9.5	377	40
OR‐5	−2.9	−9.4	1017	2.2	226	5.8	1.5	2.6	182	37
OR‐6	2.4	−4.4	1009	4.2	320	7.4	3.6	9.2	272	38
OR‐7	1.7	−2.8	1018	1.1	41	9.0	2.6	2.5	108	14
OR‐8	3.8	0.9	1004	3.3	351	8.7	5.0	1.4	31	24
OR‐9	3.1	−4.0	1013	3.5	271	6.0	2.3	1.5	83	14
OR‐10	1.5	−2.8	1022	2.6	350	8.4	2.5	1.3	58	5
CO‐1	−0.3	−3.0	1005	3.8	359	9.5	6.7	12.8	94	60
CO‐2	−2.0	−1.7	1008	5.5	13	9.5	5.6	1.7	31	19
CO‐3	2.0	−0.7	998	4.5	11	9.9	8.8	2.3	28	44
LE‐1	−3.2	−8.5	1010	3.5	323	7.6	3.7	5.7	159	78
LE‐2	−0.2	−5.8	1010	3.2	249	7.3	2.5	4.4	186	55
LE‐3	−5.1	−12.2	1020	2.6	299	3.6	1.9	3.8	214	41
LE‐4	−3.9	−12.5	1015	5.2	307	4.1	3.0	11.5	402	79
LE‐5	−6.9	−15.1	1008	5.0	292	4.5	4.0	6.4	161	85
LE‐6	1.0	−10.3	1019	5.1	312	2.7	2.4	2.6	119	22
LE‐7	0.1	−9.9	1009	5.3	237	4.0	2.7	2.0	86	51
LE‐8	3.8	−3.5	1006	5.4	305	6.8	3.2	1.9	66	26

*Note*. Types are broken down by synoptic category into overrunning (OR), coastal (CO), or lake‐effect (LE) patterns. The term “percent snow occurrences” refers to the frequency in which a single synoptic‐type results in snowfall in the region as a percentage, while “snowfall per event” is the accumulation on snow only on days in which snow is observed.

### 3.3 Analysis of snowfall by synoptic category

Averaged over the 50‐year period, the majority of snowfall in the CDW results from overrunning and Great Lakes enhanced patterns, which respectively contribute 39.2 and 37.9% of the total snowfall (Table 3). There are approximately 30 overrunning system and 28 Great Lakes enhanced snowfall events each snow season that generate 2.8–3.0 cm of snowfall per event. Both categories produce over 80 cm of snowfall individually, averaged across the watershed in a typical snow season. In contrast, coastal mid‐latitude cyclones only produce approximately 34.5 cm of snowfall each snow season, equating to just over 16% of the average total. Despite this, coastal mid‐latitude cyclones are the most impactful to the CDW on an event‐by‐event basis, where average snowfall totals per event approach 7.0 cm (Table 3).

**Table 3:** Summary snowfall statistics for each synoptic weather‐type category. Standard deviation of values given in parentheses

Synoptic category	Average snowfall (cm)	Frequency of snowfall events (days)	Snowfall per event (cm)	Contribution to total snowfall (%)
Overrunning	83.6 (31.4)	29.8 (6.5)	2.8 (0.8)	39.2
Coastal	34.5 (23.7)	5.1 (2.6)	6.9 (3.2)	16.2
Lake‐effect	80.9 (28.4)	27.9 (7.2)	3.0 (1.0)	37.9
All three	199.0 (53.7)	62.8 (10.5)	3.2 (0.7)	93.3

**Figure 5:**
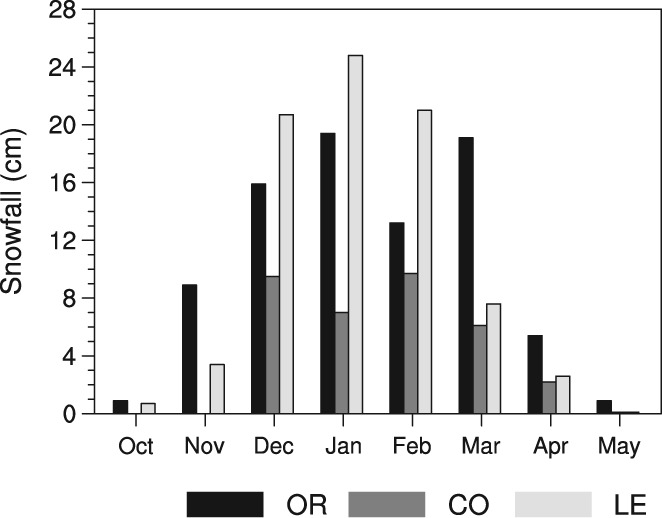
The seasonal cycle of snowfall (cm) averaged over 1960–2009 by synoptic category: inland mid‐latitude cyclones (OR; black), coastal mid‐latitude cyclone (CO; dark grey), and Great Lakes enhanced patterns (LE; light grey)

Examining variations in snowfall by synoptic category within the seasonal cycle reveals more information on their impact to the CDW. Monthly snowfall averages from overrunning systems exhibit a double peak, corresponding to January and March where each month generates approximately 20 cm of snowfall each year (Figure 5). The decline in February snowfall is likely more than a result of 10% fewer days in the month compared to January and March, but also due to a decreased frequency of the synoptic types during February. For coastal mid‐latitude cyclones, snowfall is relatively consistent across the months of December through March, with no snowfall occurring in the autumn months of October and November. For Great Lakes enhanced patterns, snowfall totals are highest during the months of December, January, and February with relatively small amounts of snow accumulating during the remaining months. While this study does not claim snowfall from Great Lakes enhanced patterns are exclusively from lake‐effect processes, the December–February peak in snowfall detected here does closely correspond to the typical timing of lake‐effect storms and snowfall associated with Lake Erie and Lake Ontario (Suriano and Leathers, 2017a; 2017b). This supports that snowfall from the Great Lakes enhanced patterns in this study is likely dominated by lake‐effect snow.

**Figure 6:**
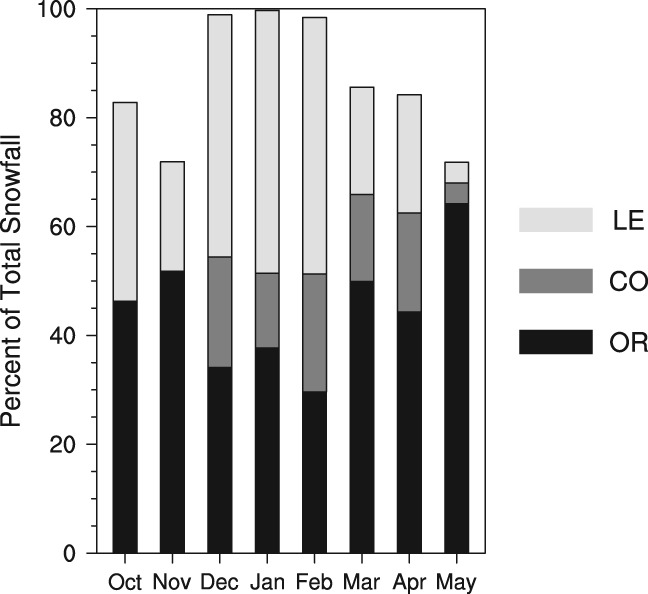
Seasonal cycle of contribution of total snowfall (%) averaged over 1960–2009 by synoptic category: inland mid‐latitude cyclones (OR; black), coastal mid‐latitude cyclone (CO; dark grey), and Great Lakes enhanced patterns (LE; light grey)

Snowfall contribution by synoptic category to total CDW snowfall also varies within the seasonal cycle (Figure 6). In the winter months (DJF), when the maximum amount of snowfall accumulates in the watershed, snowfall from Great Lakes enhanced patterns contributes the most at approximately 45–48% of the monthly totals. Outside of the winter months, snowfall from Great Lakes enhanced patterns represents less than 20% of their monthly totals, with the exception of October that experiences very little total snowfall. In the non‐winter months (non‐DJF), overrunning systems contribute more snowfall to their respective totals than the other categories combined. Snowfall from coastal mid‐latitude cyclones in December–April contributes near‐equally to the total, ranging from 14 to 22% of the monthly totals (Figure 6). Long‐term seasonal trends in snowfall, percent of total snowfall, and frequency of snowfall events are additionally examined for each synoptic category using simple linear regression; from 1960 to 2009, no statistically significant linear trends are detected.

### 3.4 Within watershed variability

While only four stations are used to generate the watershed snowfall, by examining snowfall at each of these stations individually, useful information into the spatial heterogeneity of snowfall by synoptic category can be obtained. Table 4 examines the total snowfall and contribution of total snowfall by synoptic category at the four station locations (Arkville, Delhi, Slide Mountain, and Walton). There is large spatial variability in the amount of snowfall accumulating at stations within the watershed. The Slide Mountain and Walton station locations each accumulate over 220 cm/year while the Arkville station accumulates 100 cm less snowfall per year in comparison. Arkville lies within a relatively dry portion in the central part of the region that receives less precipitation on average due to the surrounding orography that shields the location from both westerly/northwesterly flow (OR and LE patterns) and southeasterly flow (CO) systems (e.g., Thaler, 1996). Snowfall contributions by synoptic types are additionally examined. Snowfall contribution from coastal storms is the lowest for the two stations farthest from the coast (Walton and Delhi) while interestingly, the percent of snowfall from Great Lakes enhanced patterns does not decrease as the distance between the station and the Great Lakes increases. Snowfall contribution from Great Lakes enhanced patterns is very consistent spatially, ranging between 38.0 and 38.7% of individual stations’ total snowfall.

**Table 4:** Contribution to total snowfall (in %) and average seasonal snowfall (in cm) for overrunning systems, coastal storms, and lake‐effect patterns at each of the four observation stations within the CDW

	Overrunning systems	Coastal storms	Lake‐effect patterns	Total
*Contribution to total snowfall (%)*				
Arkville	36.8	19.4	38.7	94.9
Delhi	39.1	16.4	38.2	93.7
Slide Mountain	37.3	17.1	38.0	92.4
Walton	40.7	14.2	38.1	93.0
*Average seasonal snowfall* (cm)				
Arkville	48.0	25.2	50.5	123.7
Delhi	68.0	28.4	66.4	162.8
Slide Mountain	90.1	41.5	91.9	223.5
Walton	100.6	35.1	93.9	229.6

## 4 DISCUSSION

The relative breakdown of coastal mid‐latitude cyclone, overrunning systems, and Great Lakes enhanced patterns in the CDW is similar to findings from Karmosky (2007) who examined snowfall contribution from similarly classified synoptic conditions for the entire northeast United States using a 1 × 1° snowfall data set (Dyer and Mote, 2007; Kluver *et al.*, 2017). The region most closely corresponding to the CDW in the 1° data set includes observation stations that are outside the boundaries of the watershed; however, comparisons can still be drawn to this study. In the region most closely matching the CDW, overrunning storms contribute approximately 30–40%, coastal storms (coastal mid‐latitude cyclones) contribute between 20 and 25%, and lake‐effect patterns (Great Lakes enhanced) contribute 25–30% of the total snowfall according to the results in Karmosky (2007). Towey *et al.* (2018) find that extratropical cyclones contribute significantly to the largest precipitation events in this region, including cool season events in which cyclone tracks follow a meridional pattern similar to the CO type identified in this study (Figure 3b). Towey *et al.* (2018) also demonstrate that upper level support, rather than enhanced moisture availability, is the primary mechanism explaining the largest events. The 500‐hPa composite plots (Figure 4b), which show enhanced meridional flow for the CO type, also suggest increased wave energy and upper‐level support compared to the other types. While differences in methodology, station availability, and study period exist between this study and the Karmosky (2007) and Towey *et al.* (2018) studies, the results are consistent.

Due to poor data availability in the CDW, average snowfall was determined by only four stations. While the stations appear to be homogeneously distributed within the geographical boundary of the watershed, in fact it is likely that snow events at these stations are more influenced by westerly flow: three stations are located in the two most western and largest sub‐basins (Cannonsville and Pepacton); and, while the fourth station (Slide Mountain) is closer to the eastern boundary of the CDW, the station is situated at approximately 800 m in elevation on the western side of the highest ridge in the CDW. Thus, flow from the west, as is the case for Great Lakes enhanced patterns, may encounter additional topographically forced uplift and may result in higher snowfall totals at this station compared to snowfall from the other synoptic categories. These factors may partially explain the consistent nature of the contribution of total snowfall from Great Lakes enhanced patterns within the watershed, and may contribute to the higher value detected in this study compared to that shown in Karmosky (2007) that included stations outside of the CDW. Additionally, given the three sub‐basins of the CDW not represented with observation stations in the basin‐wide average are clustered on the eastern side of the watershed, it is also possible that easterly flow events are under‐represented. Such flow events could occur during a coastal cyclone and is one possible explanation to the distribution of snowfall totals across the basin.

Though care was taken in isolating synoptic weather types conducive to lake‐effect snow (Suriano and Leathers, 2017a), the authors do acknowledge snowfall from Great Lakes enhanced patterns in this study may not be exclusively snowfall generated from mesoscale lake‐effect snow bands and may consist of lake‐enhanced snowfall or small amounts of wrap‐around from mid‐latitude cyclones. However, nearly 38% of total snowfall in the CDW appears to be generated by synoptic conditions conducive to lake‐effect snow. Such a result directly contrasts conventional wisdom on the geographic reach of mesoscale lake‐effect snowfall (e.g., Blechman, 1996), and is also discussed in Hall *et al.* (2018) relative to the Catskill Mountains; this warrants further study. While significant trends in snowfall from Great Lakes enhanced synoptic weather types were not apparent, given other work examining trends in lake‐effect snowfall across New York State (i.e., Burnett *et al.*, 2003; Hartnett *et al.*, 2014), a more thorough investigation into detecting and determining the contribution of lake‐effect snowfall in the CDW will be pursued in future work.

It should also be mentioned that as synoptic weather types were qualitatively grouped into the three primary synoptic categories following the TSI procedure, user selection can influence the results. One particular synoptic type (OR‐6) contained very similar characteristics to a Great Lakes enhanced pattern, but was ultimately binned into the overrunning systems category (Table 2). Synoptic‐type OR‐6 is characterized by a 1,008 Low near coastal Maine, to the northeast of the CDW, that occurs during the spring months of March, April, and May (see Figure S1, Supporting Information for sea level pressure composite). Examining composites of sea level pressure in the preceding days (not shown), the Low tracks to the east from the central Great Lakes region into the northeast over the course of 1–1.5 days. Compared to other Great Lake enhanced patterns, the Low of OR‐6 is further south and west, closer to the CDW. As such, the CDW is within close enough proximity to the Low that it is assumed to receive relatively high amounts of “wrap‐around” snow from the cyclone for a majority of the day in which the type occurred. While almost exclusively the day(s) following an OR‐6 synoptic type are Great Lakes enhanced types, the authors elected to bin OR‐6 as an overrunning system in an attempt to limit the amount of snow accumulating due to non‐lake‐effect mechanisms in the Great Lake enhanced patterns category.

Results from the study were re‐examined with type OR‐6 included as a Great Lakes enhanced pattern for comparison purposes in justifying its inclusion in the overrunning systems category. Type OR‐6 is among the most impactful snow‐producing systems, contributing 9.5% of the total seasonal snowfall, on average. As such, when type OR‐6 is treated as a Great Lakes enhanced pattern, the amount of snow in this category increases to nearly 50% of the seasonal total, with almost all of that additional snowfall contributed by type OR‐6 occurring in March. March is not normally considered a prominent month for lake‐effect snow as the lakes are typically sufficiently frozen or in their stable season, inhibiting the necessary heat and moisture fluxes for cloud bands to form (Niziol *et al.*, 1995). Thus, it is assumed much of the snow from OR‐6 is likely not from lake‐effect/enhanced processes, justifying the decision to not consider it a Great Lakes enhanced pattern.

## 5 CONCLUSIONS AND IMPLICATIONS

This study uses a weather classification procedure to categorize snowfall in the CDW based on synoptic conditions. This region is unique in that it regularly experiences snowfall from coastal mid‐latitude cyclones, overrunning systems, and from Great Lakes enhanced patterns that collectively result in approximately 213 cm of snowfall each season when averaged across the CDW. Snowfall from Great Lakes enhanced patterns and overrunning systems contribute near‐equally to the snowfall in the CDW with each contributing approximately 81–84 cm/year, or 38–39% of the total. Coastal mid‐latitude cyclones are the least frequent of the three categories, and despite producing over twice the amount of snowfall per event compared to inland mid‐latitude cyclones and Great Lakes enhanced patterns, coastal systems produce only 16% of the CDW's total snowfall.

Within the seasonal cycle, snowfall from Great Lakes enhanced patterns dominate during the prime lake‐effect months of December through February when the eastern Great Lakes are in their unstable season (Niziol *et al.*, 1995). Overrunning systems produce snowfall over the entire October through May snow season, with average monthly snowfall totals exceeding 9 cm/month from November through March. Coastal mid‐latitude cyclones that produce substantial snowfall were not detected during autumn, but contribute relatively equally over the remaining months examined. The lack of snowfall in autumn from coastal systems is likely due to the systems being too warm to produce snowfall, where a vast majority of the precipitation falls as rain.

Snowfall magnitude, frequency, and density from different synoptic conditions is expected to change at different rates over the coming century as anthropogenic climate change continues. The conventional wisdom of warmer temperatures leading to a greater rain/snow ratio will likely lead to reduced snowfall totals from mid‐latitude cyclones in favour of enhanced rain totals. Potential changes in lake‐effect snowfall in a warmer climate are more complicated. Warmer temperatures may lead more lake‐effect rain at the expense of lake‐effect snow as surface and atmospheric temperature become too warm to support frozen hydrometeors (Notaro *et al.*, 2014; 2015; Suriano and Leathers, 2016). However, over the next two decades, some future projections indicate increasing snowfall (Suriano and Leathers, 2016). A warmer climate is expected to lead to warmer lake‐surface temperatures, potentially allowing for enhanced instability over the lakes, stronger convection, and higher snowfall totals in the short term. At the same time, warmer temperatures would also lead to less lake ice and more evaporation. Lake‐ice concentration above 70% begins to inhibit the heat and moisture fluxes necessary for lake‐effect snow development, and less lake ice would theoretically yield a greater potential for lake‐effect snow development (Gerbush *et al.*, 2008).

Suriano and Leathers (2016) also found that lake‐induced snowfall is expected to decline at a faster rate than snowfall induced by non‐lake processes after approximately 2030. Differing rates of change between snowfall‐producing conditions carry wide‐ranging implications to NYC water resources. Snowfall precipitating as rain in a warmer climate may have negative effects on the CDW due to the potential for changes in the winter versus spring runoff and increased winter reservoir storage levels (Frei *et al.*, 2002; Matonse *et al.*, 2013). Additional complications may arise due to an earlier occurrence of rain‐on‐snow ablation events in the early spring and late winter months. Such events can have a particularly devastating impact to both society and the environment where liquid precipitation and snowmelt‐generated runoff can overwhelm streams and reservoirs, and lead to flooding (e.g., Leathers *et al.*, 1998).

Snow water equivalent (SWE) is critically important from a water resources perspective, and the density of snow does vary across different snowfall‐generating systems and regionally (Ellis and Johnson, 2004; Baxter *et al.*, 2005). In this study we consider only snowfall, not SWE accumulation, and as such, further research into SWE under a variety synoptic conditions across the watershed is warranted. Future work will also investigate the contribution of total snowfall from Great Lakes enhanced patterns in greater detail, and evaluate the physical mechanisms behind the inter‐annual variability of snowfall‐producing synoptic weather types.

## Supporting information

Figure S1.
