# Supplementary figures and images for: Contribution of snowfall from diverse synoptic conditions in the Catskill/Delaware Watershed of New York State
Source: Int J Climatol. 2019 Mar 11;39(8):3608–18. doi: 10.1002/joc.6043 (PMC6921276; doi:10.1002/joc.6043)

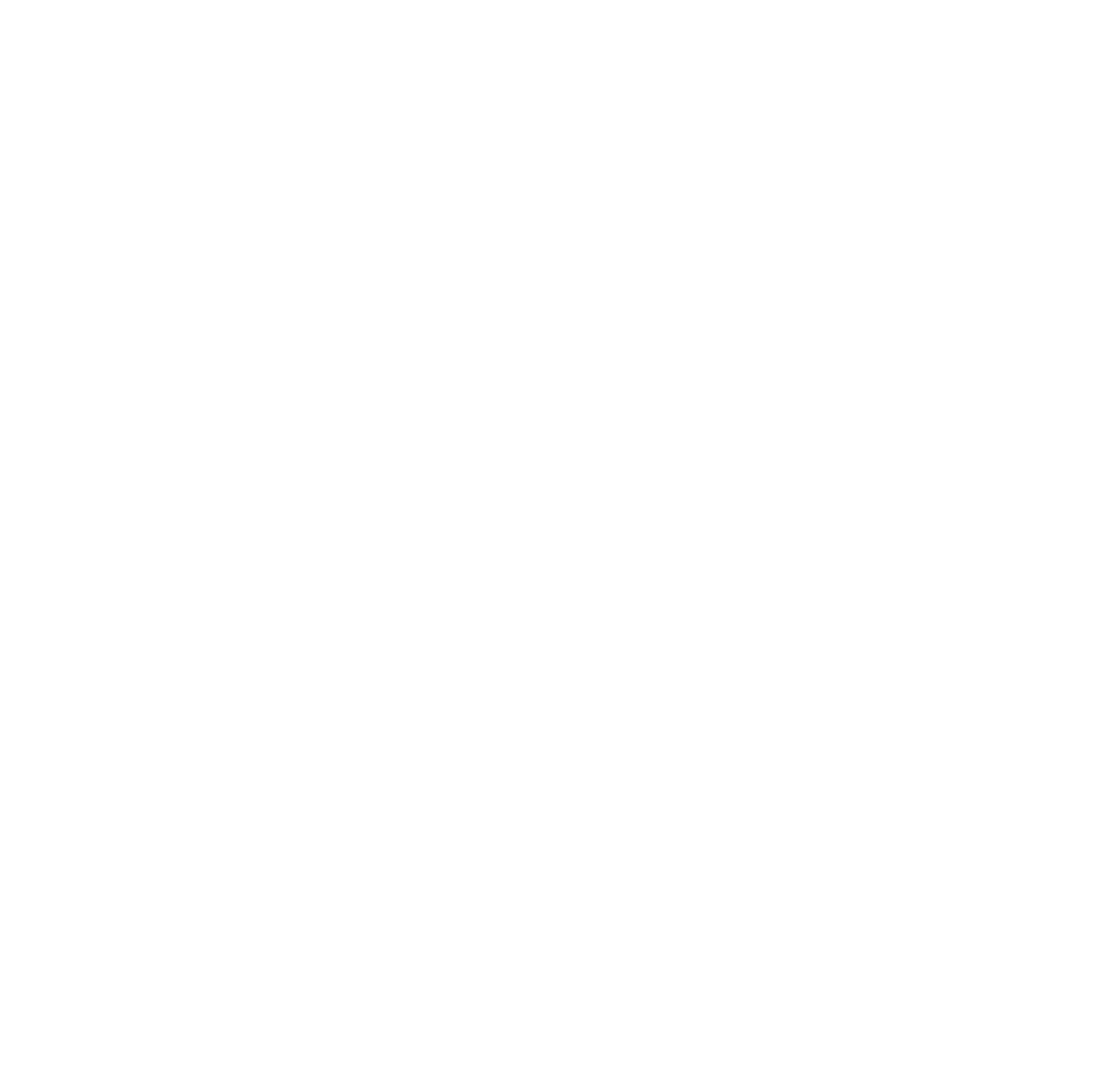

Overrunning Systems

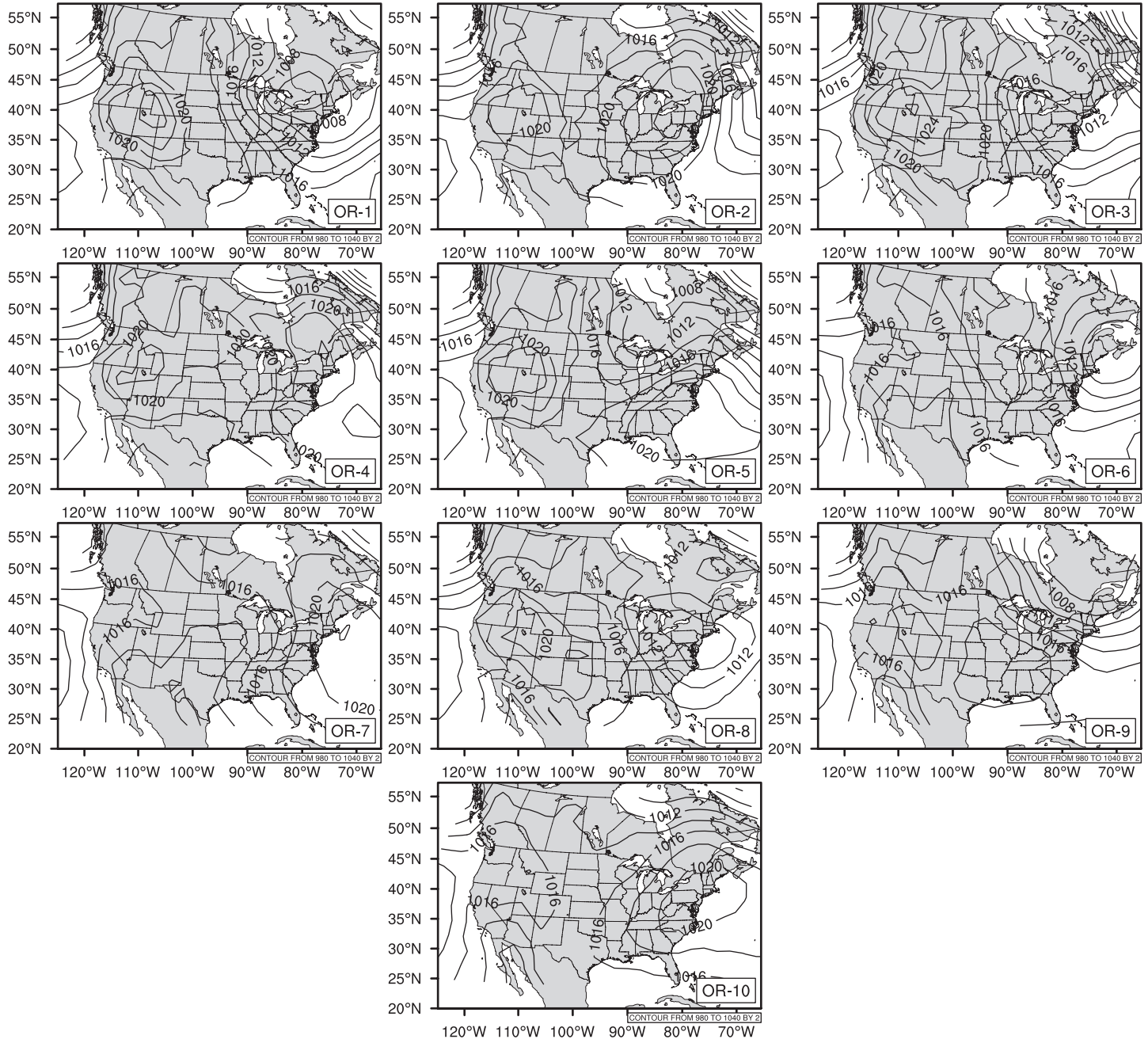

Supplement: Supplementary file 1 — Figure S1. [file JOC-39-3608-s001.pdf]
